# Rescue of 2-Deoxyglucose Side Effects by Ketogenic Diet

**DOI:** 10.3390/ijms19082462

**Published:** 2018-08-20

**Authors:** Martin Voss, Nadja I. Lorenz, Anna-Luisa Luger, Joachim P. Steinbach, Johannes Rieger, Michael W. Ronellenfitsch

**Affiliations:** 1 Dr. Senckenberg Institute of Neurooncology, University Hospital Frankfurt, Goethe University, Schleusenweg 2-16, 60528 Frankfurt am Main, Germany; nadja.lorenz@googlemail.com (N.I.L.); Anna-Luisa.Luger@kgu.de (A.-L.L.); joachim.steinbach@med.uni-frankfurt.de (J.P.S.); m.ronellenfitsch@gmx.net (M.W.R.); 2 University Cancer Center Frankfurt (UCT), University Hospital Frankfurt, Goethe University, Theodor-Stern-Kai 7, 60590 Frankfurt am Main, Germany; 3 German Cancer Consortium (DKTK), Partner Site Frankfurt/Mainz, Theodor-Stern-Kai 7, 60590 Frankfurt am Main, Germany; 4 LOEWE Center for Personalized Translational Epilepsy Research (CePTER), University Hospital Frankfurt, Goethe University, Theodor-Stern-Kai 7, 60590 Frankfurt am Main, Germany; 5 Interdisciplinary Division of Neuro-Oncology, University Hospital Tübingen, Hoppe-Seyler-Straße 3, 72076 Tübingen, Germany; j.rieger@uni-tuebingen.de

**Keywords:** 2-deoxyglucose (2-DG), isocaloric ketogenic diet, glycolysis, Warburg effect, metabolic cancer therapy

## Abstract

Cancer metabolism is characterized by extensive glucose consumption through aerobic glycolysis. No effective therapy exploiting this cancer trait has emerged so far, in part, due to the substantial side effects of the investigated drugs. In this study, we examined the side effects of a combination of isocaloric ketogenic diet (KD) with the glycolysis inhibitor 2-deoxyglucose (2-DG). Two groups of eight athymic nude mice were either fed a standard diet (SD) or a caloric unrestricted KD with a ratio of 4 g fat to 1 g protein/carbohydrate. 2-DG was investigated in commonly employed doses of 0.5 to 4 g/kg and up to 8 g/kg. Ketosis was achieved under KD (ketone bodies: SD 0.5 ± 0.14 mmol/L, KD 1.38 ± 0.28 mmol/L, *p* < 0.01). The intraperitoneal application of 4 g/kg of 2-DG caused a significant increase in blood glucose, which was not prevented by KD. Sedation after the 2-DG treatment was observed and a behavioral test of spontaneous motion showed that KD reduced the sedation by 2-DG (*p* < 0.001). A 2-DG dose escalation to 8 g/kg was lethal for 50% of the mice in the SD and for 0% of the mice in the KD group (*p* < 0.01). A long-term combination of KD and an oral 1 or 2 g 2-DG/kg was well-tolerated. In conclusion, KD reduces the sedative effects of 2-DG and dramatically increases the maximum tolerated dose of 2-DG. A continued combination of KD and anti-glycolytic therapy is feasible. This is, to our knowledge, the first demonstration of increased tolerance to glycolysis inhibition by KD.

## 1. Introduction

**Figure 1:**
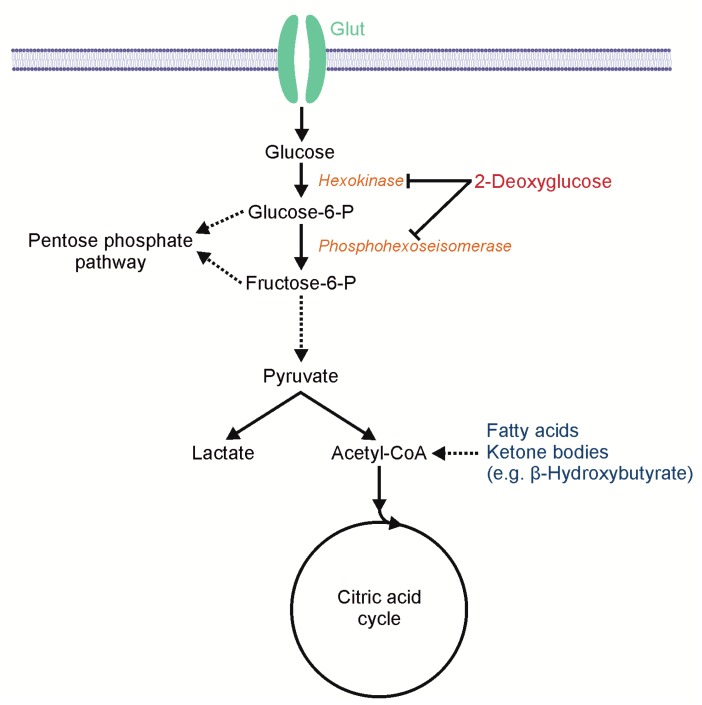
Scheme of 2-deoxyglucose (2-DG)-mediated metabolic effects. 2-deoxyglucose (2-DG) inhibits glycolysis by blocking hexokinase and phosphohexoseisomerase. Fatty acids and ketone bodies, for example β-hydroxybutyrate, enter the metabolic pathway downstream of this block and thus enable the citric acid cycle and oxidative phosphorylation.

A key feature of malignant tumors and also a hallmark of cancer is a change in metabolism [1], for example, glioblastomas (GBs), the most common malignant brain tumor in adults, and other cancers display elevated glycolysis even in the presence of oxygen (aerobic glycolysis). This effect was first described by Otto Warburg [2]. The glucose dependency of malignant tumors is also the basis of ^18^F-fluorodeoxy-glucose positron emission tomography (FDG–PET) for tumor diagnosis and staging. Potential explanations for this energetically inefficient use of glucose in cancer cells might be metabolic requirements that extend beyond the ATP production and include the need for certain precursors to generate biomass [3]. The altered cancer metabolism might expose a targetable Achilles’ heel as a selective therapeutic approach. Even though aerobic glycolysis was discovered almost a century ago, no specific treatment has since been established. To the authors’ knowledge, the first attempt to directly inhibit glycolysis in patients with a chemotherapeutic drug was 2-deoxyglucose (2-DG) in 1958 [4]. 2-DG is a structural analogue to glucose, differing from glucose only by the absence of one oxygen atom at the C2 position. Glycolysis is blocked after the intracellular phosphorylation of 2-DG by hexokinase (Figure 1), which leads to ATP depletion and the activation of AMP-activated protein kinase (AMPK) [5,6,7]. The effect of 2-DG is enhanced in hypoxia, which is a common phenomenon in GBs [8]. Furthermore, 2-DG induces the neuronal commitment of tumor cells [9], inhibits HIF-1α [10] as well as N-linked glycosylation [11], and sensitizes tumors cells to radiation therapy and chemotherapy [12]. Despite these promising effects in vitro, 2-DG as a monotherapy failed to show a significant antitumor effect in many in vivo experiments in mice and patients, and 2-DG was established as a laboratory tool but not as an anti-cancer drug. One major plausible reason for the thus far failure of 2-DG in vivo could be an insufficient dosage. 2-DG is effective if the 2-DG/glucose ratio ranges between 0.5/1 and 1/1, or even exceeding the glucose levels [6]. Studies in patients showed clinical symptoms of hypoglycemia limiting the dose to 250 mg/kg orally in combination with radiotherapy [13], or 63 mg/kg orally when combined with docetaxel [14]. The typical doses used in mice were between 0.5 g/kg and 2 g/kg, administered three times a week or once daily. Different lethal doses 50% (LD_50_) in mice have been reported depending on the mode of application, and are 2.5 g/kg for intravenous, 4.5 g/kg for intraperitoneal, and 5 g/kg for subcutaneous application (BALB/cAn X DBA/2J F1 hybrids or DBA/2J mice) [15]. In addition, LD_50_ depends on the rodents as well as on the strain used for the experiment, for example, 3.85 g/kg (male BALB/c mice, intraperitoneal) [16] and 8 g/kg (Swiss male rats, intravenous) [17]. Despite the clinical symptoms of hypoglycemia, the serum analysis shows reactive hyperglycemia after the application of 2-DG, possibly antagonizing the cytotoxic qualities [18]. Hyperglycemia is mediated by catecholamine release [19] and can be antagonized, for example, by dihydroergotamine or propranolol [20]. To enhance the antitumor effect of 2-DG, it could be required to reduce the reactive hyperglycemia and thus turn the ratio in favor of 2-DG.

Non-transformed tissues can tap various metabolic sources for ATP, while the metabolism of glioma cells is less flexible [21]. We hypothesized that the preconditioning of untransformed tissues, especially neural cells with alternative energy sources, could reduce the clinical symptoms of the inhibition of cellular glucose utilization and allow higher doses of 2-DG. The ketogenic diet (KD) has the potential to achieve both tasks. The classic KD is a high-fat and low-carbohydrate diet raising serum levels of the ketone bodies and lowering the brain glucose uptake. Our group has recently shown that a KD is safe in mice and humans with glioblastoma [21,22]. The aim of this study was to determine the side effects of a combined anti-glycolytic therapy with 2-DG and calorically-unrestricted KD.

## 2. Results

### 2.1. Effects of 2-DG on Glucose Consumption and AMP-Kinase Signaling in Human Glioblastoma Cells

**Figure 2:**
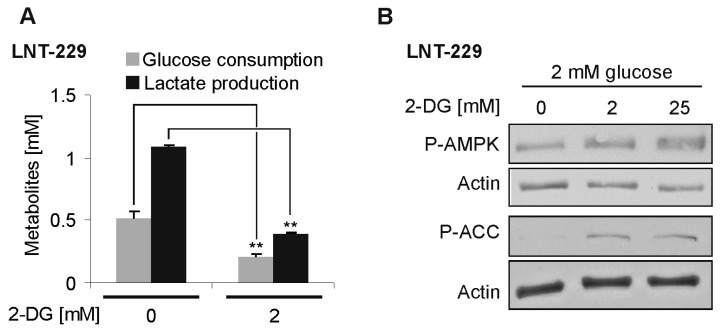
2-deoxyglucose-mediated effects on glucose consumption and AMP-kinase signaling. (**A**) Human LNT-229 glioblastoma cells were incubated in serum-free Dulbecco’s modified eagle medium (DMEM) containing 2 mM glucose in the presence or absence of additional 2-DG for 6 h, as indicated. Glucose consumption and lactate production were measured in the supernatant (*n* = 3, mean + standard deviation, ** *p* < 0.01, Student’s *t*-test). (**B**) LNT-229 cells were incubated as in (**A**), with different concentrations of 2-DG, as indicated. Cellular lysates were analyzed by immunoblot with antibodies for P-AMPK, phosphorylation target acetyl-CoA carboxylase (P-ACC), and actin.

To confirm the 2-DG-mediated effects on glucose metabolism, we treated human LNT–229 glioblastoma cells with 2-DG. The glucose consumption and lactate production were reduced in the presence of 2-DG (Figure 2A). Additionally, 2-DG activated the central nutrient-deprivation sensitive AMPK signaling pathway, as evidenced by an increase in both the phosphorylated AMPK and its phosphorylation target acetyl-CoA carboxylase (ACC) in the presence of 2-DG (Figure 2B).

### 2.2. Metabolic Alterations Induced by Ketogenic Diet in Athymic Mice (Foxn1nu)

**Figure 3:**
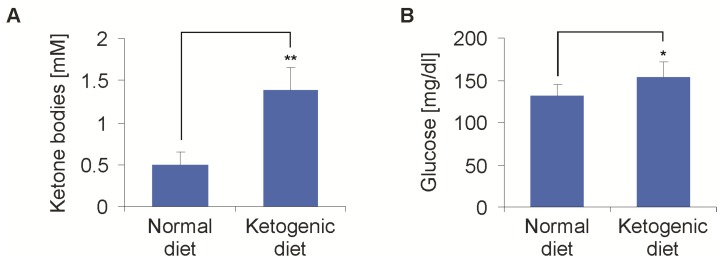
Effects of a ketogenic diet (KD) on blood ketone bodies and glucose levels. Mice were fed either a normal diet or ketogenic diet. (**A**) After one week, the blood ketone bodies showed an average of 1.38 ± 0.28 mmol/L in the KD group compared to 0.5 ± 0.14 mmol/L in the standard diet (SD) group (*n* = 8, mean + standard deviation, ** *p* < 0.01, Student’s *t*-test). (**B**) At the same time, the blood glucose was higher in the KD than in the SD group (153 ± 18 mg/dL vs. 131 ± 14 mg/dL, *n* = 8, mean + standard deviation, * *p* = 0.03, Student’s *t*-test). The measurement of the baseline blood glucose in the further course of time never showed a difference in the blood glucose between the two groups again.

In a first experiment, two groups of eight mice were fed either a standard diet (SD) or caloric-unrestricted KD, in order to establish the effects on ketosis and blood glucose. After one week, the blood ketone bodies showed an average of 1.38 ± 0.28 mmol/L in the KD group, compared to 0.5 ± 0.14 mmol/L in the SD group (*p* < 0.01) (Figure 3A). This was in line with our previous results [21]. The first measurement of the baseline blood glucose after one week of the diet was higher in the KD than in the SD group (153 ± 18 mg/dL vs. 131 ± 14 mg/dL, *p* = 0.03) (Figure 3B). The following measurements of the baseline blood glucose failed to show any differences over the course of the experiments. Overnight fasting has been added to the KD in clinical trials. In a separate experiment, the overnight fasting in addition to the KD further increased the ketone body levels (baseline: 0.7 ± 0.1 vs. 1.9 ± 0.2 mmol/L, *p* < 0.01). The combination of KD and overnight fasting led to a reduction of the blood glucose levels, with lower values in the KD group (KD + fasting 90 ± 30 mg/dL, SD 121 ± 15 mg/dL, *p* < 0.01). Fasting did not influence the metabolic responses following 2-DG administration (i.e., an increase in blood glucose). Therefore, further experiments were done without fasting.

### 2.3. Modulation of 2-Deoxyglucose-Induced Hyperglycemia by Ketogenic Diet

**Figure 4:**
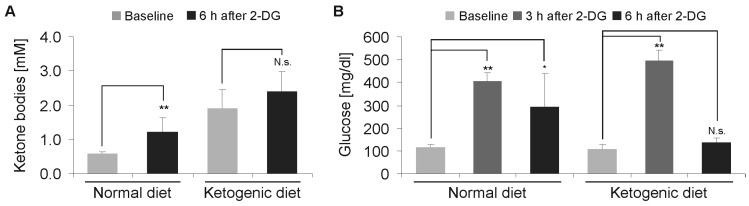
Effects of 2-deoxyglucose on blood ketone bodies and glucose levels. (**A**) Mice were fed either normal diet or ketogenic diet. Following intraperitoneal injection of 4 g 2-DG, the blood ketone bodies were determined after 6 h (*n* = 8, mean + standard deviation, not significant (N.s.) *p* > 0.05, ** *p* < 0.01, Student’s *t*-test). (**B**) Mice were fed either a normal diet or ketogenic diet. Following the intraperitoneal injection of 4 g 2-DG, the blood glucose was determined after 3 and 6 h (*n* = 8, mean + standard deviation, N.s. *p* > 0.05, * *p* < 0.05, ** *p* < 0.01, Student’s *t*-test).

To reproduce the acute toxicity of 2-DG in the low and established doses, we administered doses of 2-DG starting at 0.5 g/kg, with a subsequent increase of up to 4 g/kg by intraperitoneal injection in cohorts of eight mice. In the cohort, 4 g/kg 2-DG was not lethal for any of the mice. The blood glucose was measured after 3 and 6 h, and the ketone bodies after 6 h. We detected a significant increase in the ketone bodies in comparison to the baseline measurement in the SD group (*p* < 0.01), while a further increase in the KD group was not significant (*p* = 0.13) (Figure 4A). The blood glucose levels peaked after 3 h and were elevated after 3 h and 6 h in the SD group. In the KD group, there was a similar increase after 3 h, however, after 6 h, the levels had already returned to baseline (Figure 4B).

### 2.4. Rescue of 2-Deoxyglucose-Mediated Motion/Behavioral Constraints by Ketogenic Diet

**Figure 5:**
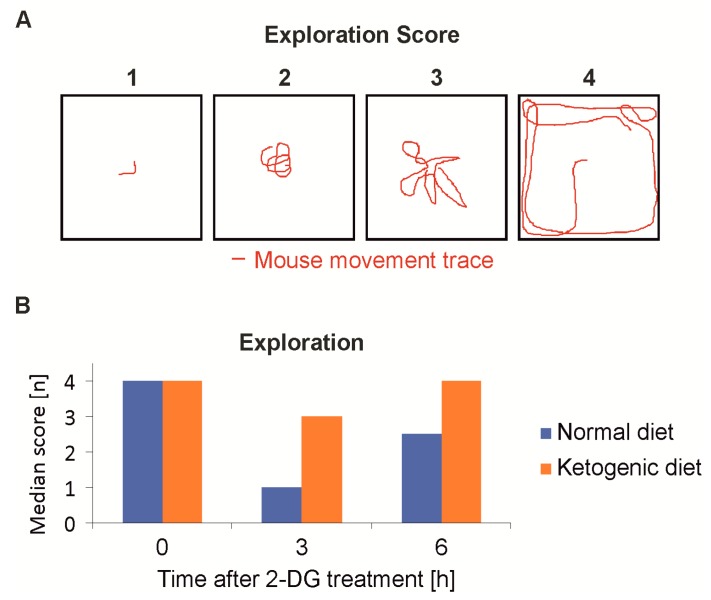
Mouse behavioral assessment after 2-deoxyglucoe treatment. (**A**) The behavior was assessed semi-quantitatively. Mice were placed in the middle of a transparent cube of 30 cm side length for one minute and the movement was recorded for later analysis. (**B**) Mice were fed either normal diet or ketogenic diet. Following intraperitoneal injection of 4 g/kg 2-DG, the level of sedation as the main side effect of 2-DG was examined by the standardized evaluation of the exploration behavior. The mice fed the ketogenic diet were less sedated and recovered faster.

At the same time points behavioral testing was performed to evaluate the clinical symptoms, which primarily manifested as sedation and reduced motion. An open-space, spontaneous motion test [23] (Figure 5A) showed that the mice of the KD group were significantly less sedated after 3 h (*n* = 8, *p* < 0.001) and had completely recovered after 6 h. The SD-fed group showed sustained sedation even 6 h post injection of 2-DG (Figure 5B). A comparison of the two treatment groups was also done employing the beam walking, however the balancing over a stick of a 6 mm diameter proved to be too difficult for even the slightly sedated mice and the results were not statistically significant (data not shown).

### 2.5. Ketogenic Diet Increases the Maximum-Tolerated Dosage of 2-Deoxyglucose

**Figure 6:**
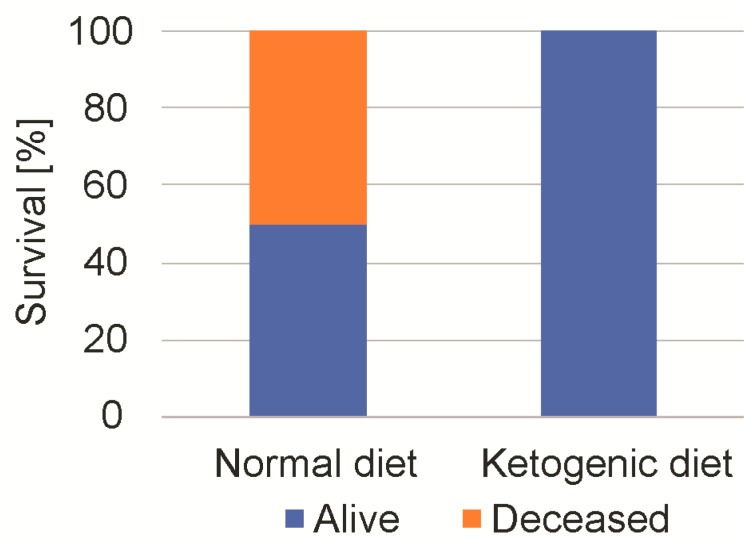
Mouse 2-deoxyglucose tolerance depending on diet. Mice were fed either normal diet or ketogenic diet. Following intraperitoneal injection of 8 g 2-DG, 50% of the mice that were fed the standard diet died, while none that were fed the KD diet died. All of the surviving mice showed a marked initial sedation and recovered without a permanent neurological deficit.

We next studied the maximum tolerable dose of 2-DG. As all of the mice had tolerated a 4 g/kg dosage, we escalated 2-DG stepwise up to 8 g/kg (administration by intraperitoneal injection) [17]. After the injection of 8 g/kg, 50% of the SD-fed mice died, while no mouse of the KD group died (*n* = 8, Chi square test, *p* < 0.01) (Figure 6). All of the mice, regardless of diet, showed a pronounced sedation for up to 6 h. The surviving mice fully recovered and showed no persistent neurological or behavioral deficit. The measurement of blood glucose after one hour showed an increase from 124 ± 14 mg/dL to 358 ±3 4 mg/dL in the SD-fed group, and from 138 ± 25 mg/dL to 342 ± 43 mg/dL in the KD-fed mice.

### 2.6. A Continuous Combined Anti-Glycolytic Therapy is Feasible

**Figure 7:**
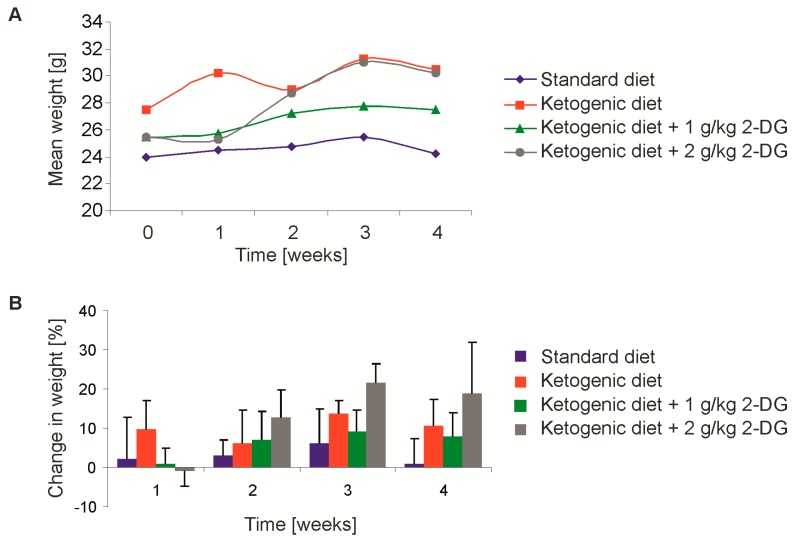
Effects on mouse weight of 2-deoxyglucose and ketogenic diet. The mice were randomized in groups of four mice and fed either a standard diet, calorically-unrestricted ketogenic diet (KD), KD with additional 1 g/kg 2-DG per oral gavage (three/week), or KD with additional 2 g/kg 2-DG (three/week). The combination of KD and 2-DG was well tolerated and we observed no change in the general behavior. (**A**) Over one month, a slight, non-significant increase in total body weight in the ketogenic groups (*p* = 0.06, Student’s *t*-test) was detected. (**B**) Change in body weight of mice relative to the start of the dietary intervention (*n* = 4, mean + SD).

To study the feasibility of a continuous combined anti-glycolytic therapy, the mice were randomized in groups of four mice and were fed either a SD, calorically-unrestricted KD, KD with additional 1 g/kg 2-DG per oral gavage (three/week), or KD with additional 2 g/kg 2-DG (three/week). The ketone bodies after one week averaged at 1.2 ± 0.18 mmol/L in the KD groups, compared to 0.4 ± 0.19 mmol/L in the SD group. The level of ketone bodies did not change with the increasing duration of the diet. There was no significant difference when 2-DG was added (*p* = 0.14). Sedation manifested with reduced motion approximately 15 to 30 min post application of 2-DG. All of the mice recovered within a 3 h period. Apart from these acute effects, the combination of KD and 2-DG was well tolerated and we observed no change in the general behavior. Over one month, a slight, non-significant increase in body weight in the ketogenic groups (*p* = 0.06) was detected (Figure 7A,B).

## 3. Discussion

Dose limitations due to toxicity are likely one reason for the lack of efficacy of antiglycolytic therapies. To fully exploit the potential of the antiglycolytic approaches in glucose-addicted cancer cells, a maximum dose and selectivity will be essential. In a model system with LNT–229 glioblastoma cells, 2-DG reduced the glucose consumption and triggered the signaling of the nutrient-deprivation sensitive AMPK pathway (Figure 2). In the mouse experiments, an isocaloric KD increased the tolerance for 2-DG, most likely by a metabolic adaptation to alternative fuels distinct from glucose. The KD preconditioning resulted in an improved functional testing and reduced death rate, when escalating the doses of 2-DG. KD failed to prevent reactive hyperglycemia after the injection of 2-DG. Horton and colleagues pretreated the mice with 0.1 mg propranolol to prevent catecholamine release-mediated hyperglycemia, before the administration of 3 g/kg 2-DG [20]. Despite a prevention of rise of blood glucose levels, they still reported a marked sedation of the animals and even death. The acute toxicity/lethality of 2-DG therefore appears not to be mediated by an excessive increase of blood glucose levels. This is in line with our results. We postulate that the intracellular block of glycolysis (leading to intracellular hypoglycemia and transient energy shortage) in the neurons is responsible for the sedative side effects, and death most likely results from a comatose sedation without sufficient brain stem reflexes and not from a hyperglycemic coma-mediated complication. The sensitivity of the neurons to glycolytic inhibition can be reduced by preconditioning to ketone bodies as an alternative energy source that neurons can readily tap [21]. A remarkable example of this ability of neurons to rely on ketone bodies is the rare glucose transporter type 1 (GLUT1) deficiency syndrome. Because of a mutation in the SLC2A1 gene, GLUT 1 is lacking, causing a severe developmental delay, intellectual disability, and other neurological symptoms [24]. The treatment consists of a KD or modified Atkins diet, to supply an alternative source of energy [25]. To our knowledge, we here report the first study demonstrating a reduction of the side effects of an inhibitor of glycolysis by preconditioning with a KD. We used 2-DG as the oldest anti-glycolytic drug. Even though a glycolysis-targeting therapy is a promising and innovative approach for cancer treatment, the severe side effects of the glycolysis inhibition limit the potential exploration in trials. For example, a trial using the glycolysis inhibitor dichloroacetate had to be stopped after almost all of the participants developed a toxic polyneuropathy [26]. In combination with KD, a rechallenge of the glycolysis inhibitors to treat cancer in humans might be feasible.

In this regard, the next step to evaluate the antitumor efficiency of the combination of KD and 2-DG should be testing in the appropriate mouse tumor models. Marsh et al. reported a promising synergistic effect of 2-DG, in combination with KD [27]. While we used an unrestricted, isocaloric, KD with high doses of 2-DG, Marsh et al. used a caloric restricted diet (20% weight loss) with a low dose of 25 mg/kg 2-DG. While a continuous caloric restricted diet is tolerated in rodents, this will not be likely in the terminally-ill patients. A calorically-restricted diet would cause a chronic weight loss and possible weaken an already frail group of mostly elderly patients. The isocaloric KD is well-tolerated by cancer patients [22,28] and could cause a chronic stress to the tumor cells. The cyclic application of high amounts of 2-DG could cause an acute additional antitumor effect and prevent tumor cell adaptation, as proposed by Seyfried et al. [29]. A model tumor, where KD and antiglycolytic therapy might have potential, is GB, the most common malignant brain tumor in adults [30]. GB is characterized by hypoxia, which can lead to resistance to chemotherapeutic drugs, together with the metabolic adaptation of tumor cells [31]. Several clinic trials evaluated bevacizumab, a humanized antibody, against vascular endothelial growth factor (VEGF), to enhance hypoxia by blocking neoangiogenesis, and thus killing tumor cells [32]. However, every trial failed to show a benefit in the overall survival [33,34]. The development of a more glycolytic phenotype as an escape mechanism has been postulated in bevacizumab treated tumor cells [35]. Our group reported that in an orthotopic mouse xenograft model, glioma with impaired oxidative phosphorylation were resistant to treatment with bevacizumab, and treatment with 2-DG re-established sensitivity to bevacizumab [36]. Therefore, antagonizing glycolysis and neoangiogenesis together could be a promising therapeutic concept.

## 4. Materials and Methods

### 4.1. Reagents, Cell Lines, and Culture Conditions

2-DG was purchased from Cayman Chemical (Biomol, Hamburg, Germany). LNT-229 cells were a kind gift of Dr. N de Tribolet (Lausanne, Switzerland) [37] and were cultured in high glucose Dulbecco’s modified eagle medium (DMEM) (4.5 g/L corresponding to 24.975 mM glucose) containing 10% foetal calf serum (FCS) (Biochrom KG, Berlin, Germany), 100 IU/mL penicillin, and 100 µg/mL streptomycin (Life Technologies, Karlsruhe, Germany) at 37 °C in a cell culture incubator (Binder, Tuttlingen, Germany) under a CO_2_ atmosphere (5%). The experiments were performed in serum free DMEM, with 2 mM of glucose and an incubation time of 6 h.

### 4.2. Measurement of Glucose and Lactate

The cell-free supernatant was collected and the glucose and lactate concentrations were measured using the biochemistry analyzer Hitachi 917 [37].

### 4.3. Lysate Preparation and Immunoblot Analysis

The procedure was performed, as previously described, following a standard immunoblotting protocol [38]. After incubation, the LNT-229 cells were washed with ice-cold phosphate *buffered* saline (PBS) and pelleted. The lysates were prepared using a lysis buffer, P, with the addition of 1% Halt™ Protease and Phosphatase Inhibitor Single-Use Cocktail (Thermo Fisher Scientific, Hamburg, Germany), diluted in a Laemmli buffer and subjected to sodium dodecyl sulfate polyacrylamide gel electrophoresis (SDS-PAGE) analysis. Subsequently, the proteins were blotted to nitrocellulose membranes (0.45 µm; GE Healthcare, Little Chalfont, UK). The membranes were probed with antibodies to phosphorylated (p)-AMPK (Thr172) (1:1000; #2531 Cell Signaling Technology, Danvers, MA, USA), p-ACC (Ser79) (1:1000; #3661 Cell Signaling Technology, Danvers, MA, USA) and actin (#sc-1616, 1:2000; Santa Cruz Biotechnology, Santa Cruz, CA, USA). The secondary anti-rabbit and anti-goat antibodies were purchased from Santa Cruz Biotechnology. Enhanced chemiluminescence was used for detection [37].

### 4.4. Animals

Six-week-old female athymic nude Foxn1nu mice (Harlan, Indianapolis, IN, USA) were used in all of the animal experiments. The local regulations were observed and approval was obtained from the local governmental authorities (Regierungspräsidium Darmstadt, approval number FK/1038).

### 4.5. Dietary Interventions

All of the animal work was performed in accordance with the National Institutes of Health Guidelines Guide for the Care and Use of Laboratory Animals and institutional standards. The mice were maintained in groups of a maximum four animals per cage, in a pathogen-free environment. On day 0 of the experiment, the animals were randomized into two diet groups, a SD rich in carbohydrates versus an isocaloric KD. The SD contained 45% carbohydrates, 41% protein, and 14% fat (ssniff Spezialdiäten GmbH, Soest, Germany). The KD was KetoCal 4:1, baked as cookies (Nutricia GmbH, Erlangen, Germany). The content of the diets has been described before [21].

### 4.6. Ketone Body and Glucose Measurement

Ketosis was measured at least once a week with Precision Xceed, test strip FreeStyle Precision Beta-Ketone (Abbott Diabetes Care Ltd., Witney, UK), starting one week after the randomization. The blood glucose level was measured using Accu-Check Performa, test strip Accu-Check Inform II (Roche).

### 4.7. Behavioral Assessment

The behavior was assessed semi-quantitatively, as established by a research group of our facility [23]. The mice were placed in an experimental setting for one minute and a video was recorded for later analysis. The mice had not been trained before.

Beam walking test: We placed the mice carefully on a bar of wood (6 mm diameter, 50 cm length), 20 cm above the ground, until they attained a firm grip. The following score was used: score 1—mouse is heavily sedated and cannot hold onto bar; Score 2—mouse is sedated, can hold onto bar for one minute but does not move; Score 3—mouse explores on bar but appears lightly sedated and does not reach the end of the beam; and Score 4—mouse moves freely on bar and reaches the end of the bar.

Spontaneous motion activity: The mice were placed in the middle of a transparent cube of 30 cm side length. The following score was used: 1—mouse is heavily sedated and does not move; 2—mouse is sedated, shows little exploration on the spot but does not move into any direction; 3—mouse explores the cube, appears lightly sedated and typically does not reach the boundary of the cube; and 4—mouse moves freely in the cube and reaches a boundary at least once.

### 4.8. Statistical Analyses

*T*-test and chi squared test were performed in Microsoft Excel for statistical analysis.
